# Algicidal Efficiency and Genotoxic Effects of *Phanerochaete chrysosporium* against *Microcystis aeruginosa*

**DOI:** 10.3390/ijerph17114029

**Published:** 2020-06-05

**Authors:** Guoming Zeng, Maolan Zhang, Pei Gao, Jiale Wang, Da Sun

**Affiliations:** 1 Chongqing Engineering Laboratory of Nano/Micro Biological Medicine Detection Technology, School of Architecture and Engineering, Chongqing University of Science and Technology, Chongqing 401331, China; 2017015@cqust.edu.cn (G.Z.); zml@cqu.edu.cn (M.Z.); 2020005@cqust.edu.cn (P.G.); 2019048@cqust.edu.cn (J.W.); 2 Institute of Life Sciences & Biomedicine Collaborative Innovation Center, Wenzhou University, Wenzhou 325035, China

**Keywords:** eutrophication, algicidal ability, genotoxic effects, *P. chrysosporium*

## Abstract

Eutrophication has become a severe environmental problem. This study evaluated the algicidal efficiency and genotoxic effects of *Microcystis aeruginosa* co-cultured with *Phanerochaete chrysosporium* for 48 h under the optimum conditions of 250 mg/L of *P. chrysosporium* at 25 °C with dissolved oxygen content of 7.0 mg/L. The results showed that the activity of algal dehydrogenase, superoxide dismutase, and peroxidase were all decreased and the malondialdehyde content increased after co-culturing. Fourier transform infrared spectroscopy and scanning electron microscopy observations showed that the functional group and structure of algal cells were significantly changed. Compared with those of control tadpoles, blood cells of *Fejervarya multistriata* tadpoles had increased micronucleus frequency (from 1.05 ± 0.09 to 1.99 ± 0.05) and abnormal nuclei (from 2.45 ± 0.06 to 5.83 ± 0.07). The tail length of *M. aeruginosa* co-cultured with *P. chrysosporium* increased from 1.12 ± 0.21 to 21.68 ± 0.34, and the comet length increased from 6.45 ± 0.09 to 36.45 ± 0.67 within 48 h. Micronucleus assay and Comet assay results demonstrated that *P. chrysosporium* might effectively remove algae and reduce genotoxic effects and may be safe for aquatic ecosystems.

## 1. Introduction

Eutrophication results from the enrichment with nitrogen, phosphorus, and other nutrients, and may cause abnormal reproduction in algae and other aquatic plants, decreased water transparency and dissolved oxygen content, poor water quality, and death of aquatic organisms [1]. It has become a global environmental problem and threatens to destroy the balance of aquatic ecosystems such as lakes, reservoirs, and closed and semi-enclosed rivers [2,3].

*Microcystis aeruginosa* is a common algae involved in water eutrophication. Its growth, metabolism and death will not only pose a serious threat to the aquatic environment and ecological security, and have an inestimable impact on human survival and health [4]. Therefore, treating *M. aeruginosa* has important social and practical significance. Three methodologies are commonly used in the removal of algae and algal toxins: Physical, chemical, and biological methods. The physical method is not comprehensive or cost effective, and the chemical methods are prone to secondary pollution and poor security. However, biological remediation methods are relatively cost effective and have low risk when compared with the physical and chemical methods; making them promising candidates for future research [5]. White-rot fungi can degrade or decrease various environmental pollutants, including dyes, lignin, and toxic wastes [6]. Previous studies indicated that their suppressive effect on algal species could be a result of enzymes secreted during incubation, such as laccase [7,8,9,10,11]. However, the mechanisms underlying the ecotoxicological relationships between white-rot fungi and algae still need to be studied.

Modern molecular biology methods provide a new avenue for investigating ecological toxicology mechanisms at a cellular and molecular level [12,13]. A variety and combination of physical and chemical factors detrimentally affect the structure and function of DNA, resulting in genetic diseases, cell mutation, and ultimately death [14]. Comet assays can quantitatively determine DNA integrity at the single-cell level are suitable proxies for monitoring genetic damage [15,16,17]. The micronucleus is an abnormal structure and chromosome aberration in eukaryotes [18,19,20]. Pollution can be monitored by observing the cell micronuclei of plant and animal tissue. In recent years, it has been widely used in radiation injury, chemical mutagenesis, and environmental monitoring [21,22,23].

Some algae-lysing microorganisms of mature or artificial domestication may produce endotoxins or exotoxins during the process of algal cell metabolism or death, which poses a severe threat to a body of water. The algicidal efficiency and toxicity of the algae-control system of white- rot fungi need to be determined for it to be safely applied to the treatment of eutrophic water. In this study, we evaluated the genotoxic effects of *Phanerochaete chrysosporium*, a typical white-rot fungi, on *M. aeruginosa* in *Fejervarya multistriata tadpoles* by a comet and micronucleus test. Structural characteristics, including the algal cell and surface morphology, were also assessed and compared using Fourier transform infrared spectroscopy (FTIR) and scanning electron microscopy (SEM) to investigate the algicidal efficiency of *P. chrysosporium*.

## 2. Materials and Methods

### 2.1. Algal Strains and Cultivation

*M. aeruginosa*, which as research target, was provided by the Freshwater Algae Culture Collection of the Chinese Academy of Sciences. All stock and experimental cultures were maintained at 25 °C under a 12:12 h (light:dark) cycle at approximately 90 µmol photons m^−2^·s^−1^ provided by cool-white fluorescent lamps to achieve exponential growth. The growth medium for *M. aeruginosa* was BG-11 and 1000 mL distilled water.

### 2.2. Fungal Strains

*P. chrysosporium* was provided by the Institute of Microbiology (China). The strain was maintained on potato dextrose agar (PDA) plates and cultured at 35 ℃ for 5 days. It was then used in further experiments.

### 2.3. Animal and Experimental Design

One hundred of *F. multistriata* tadpoles, which were used for toxicology testing, were obtained from a Chongqing farmland suburb and then placed in a non-polluted eco-pond (27–28 °C). The healthy tadpoles were of average uniform size at 32–36 mm, with an average body length and weight of 26.28 ± 0.34 mm and 0.19 ± 0.019 g, respectively. 

*F. multistriata* tadpoles were fed to *M. Aeruginosa.* The *M. aeruginosa* was treated with *P. chrysosporium* under the optimum conditions of 250 mg/L *P. chrysosporium* at dissolved oxygen content of 7.0 mg/L at 25 °C and 12:12 h (light:dark) cycle for 24 h and 48 h. Control cultures were maintained concurrently throughout the experiment at identical conditions in the absence of *P. chrysosporium* and no feeding during the experiment. All experiments were conducted in triplicate for the micronucleus assay and the comet assay.

### 2.4. Chlorophyll—A Content Test

The algicidal tests were conducted under the conditions of 25 °C, pH 7.0, 7.0·mg·L^−1^ DO, and 12:12 h (light:dark) cycle. All experiments were conducted in triplicate. The total chlorophyll-a was extracted with 90% acetone and quantified using the repeated freezing–thawing extraction method [24].

### 2.5. Antioxidant Enzyme Assays

Aliquots of 25 µL of the samples were collected and centrifuged at 8000 rpm for 20 min for enzyme activity tests. The activities of dehydrogenase (DHA), superoxide dismutase (SOD), and peroxidase (POD) in the supernatant were determined based on the triphenyl tetrazolium chloride- dehydrogenase reduction [25], nitrogen blue tetrazole [26], and the guaiacol method [27], respectively. The content of malondialdehyde (MDA) was measured using the thiobarbituric acid method [28].

Fourier transform infrared spectroscopy disks were prepared by mixing 2 mg of lyophilized sample using a freeze-dry system (FREEZONE-6, Hitachi, Japan) with 150 mg of KBr. The samples were then pressed in a standard device using a pressure of 16 MPa to produce 13-mm-diameter pellets. Fourier transform infrared (FTIR) spectra were recorded between 4000 and 400 cm^−1^ using a Spectrum GX (Perkin Elmer, Waters, US).

### 2.6. Scanning Electron Microscopy

The microstructural changes and surface characteristics of the *M. aeruginosa* and *M. aeruginosa* treated with *P. chrysosporium* were tested through a SEM TESCAN VEGA II LMU (COXEM, Hitachi, Japan) at 20 kV accelerating voltage.

### 2.7. Micronucleus Assay

The surfaces of the tadpoles were dried with filter paper, the tails were cut, and then the tail blood was used to make a blood smear, which was dried naturally and fixed with methanol for 15 min. Wright’s stain was applied for 15–20 min, followed by washing with phosphate-buffered saline (PBS) (pH 6.8). Finally, the blood smears were dyed with the Gimsa solution for 25 min, washed with deionized water, and dried naturally for observation under a microscope. For each blood smear, 1000 cells were observed, and the number of micronuclei and abnormal red blood cells was recorded. The micronucleus cell rate (MCN) = number of cells with micronucleus/total number of observed cells * 1000‰, and the nuclear abnormal cell rate (ONA) = total number of cells with nuclear abnormalities (except micronuclei)/total number of observed cells * 1000‰. The data obtained from the experiment, the comparison between the control group and the treatment group were analyzed using the Origin 8.0 (Originlab, US) software method. All experiments were conducted in triplicate.

### 2.8. Comet Assay

The heads of the *F. multistriata* tadpoles were cut; blood cells were collected and diluted with PBS (pH 6.8) until the cell density reached 10^5^–10^6^ mL. These blood cells were used directly for the comet assay. The buffer containing the blood cells was transferred into a 2-mL centrifuge tube. Completely frosted microscopic slides were covered by a 90 µL thin layer of 0.7% normal melting agarose (NMA), (NMA), which was allowed to solidify at 4 °C for 30 min. Then, 75 µL of low melting point agarose (1%) containing 25 µL of blood cells was added to cover the NMA at 4 °C for 30 min. The slides were placed in a lysis solution at 4 °C (2.5 M NaCl, 100 mM Na_2_EDTA, 10 mM Tris-HCl, 1% Triton X-100 and 10% DMSO, pH 10) for 1.5 h in dark. Then, the slides were washed with chilled distilled water and placed on a horizontal gel electrophoresis apparatus in an alkaline buffer (300 mM NaOH, and 1 mM Na_2_EDTA, pH > 13) at 20 °C (20 V and 200 mA) for 30 min in the dark to avoid DNA damage. After electrophoresis, each slide was neutralized with the neutralization buffer (0.4 M Tris-HCI, pH 7.3) three times, washed with chilled distilled water, and placed in ethanol for 5 min at 4 °C. Finally, the slides were stained with Godview for 20 min in the dark and observed under a fluorescence microscope at 490 nm, with the excitation plate wavelength at 420–485 nm, and the emission plate wavelength at 515 nm [27]. Each group contained three parallel samples, and 100 cells were observed for each slide; the images of comets were analyzed using the CASP 10.0 (NIH, US) software. 

### 2.9. Statistical Analysis

The data obtained from the experiment were analyzed and processed using the Origin 8.0 software processing system and Windows Excel and Word (2003, 2010 version) Office software.

## 3. Results and Discussion

### 3.1. Algicidal Efficiency

**Figure 1:**
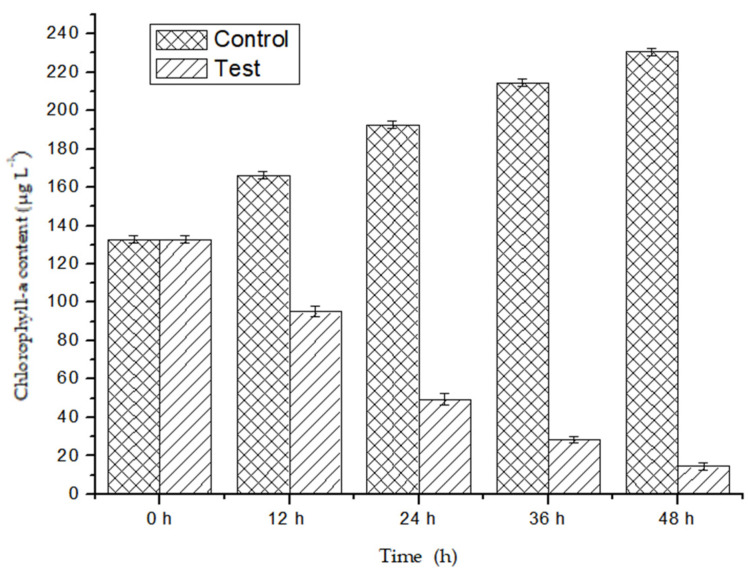
Algicidal efficiency of *Phanerochaete chrysosporium*: *Microcystis aeruginosa* alone and *M. aeruginosa* treated by *P. chrysosporium*. Data are expressed as the mean ± SD (*n* = 3).

**Figure 2:**
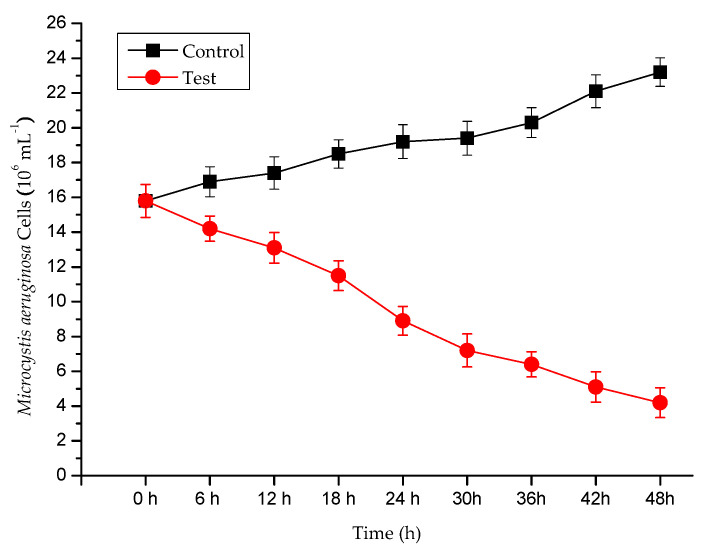
The growth curves of *M. aeruginosa* Cells of *P. chrysosporium*: *M. aeruginosa* alone and *M. aeruginosa* treated by *P. chrysosporium*. Data are expressed as the mean ± SD (*n* = 3).

The chlorophyll-a content of *M. aeruginosa* was evaluated after the *P. chrysosporium* treatment. *P. chrysosporium* induced reductions of 91.23% in the chlorophyll-a content of *M. aeruginosa* within 48 h (Figure 1), indicating *P. chrysosporium* limits growth effectively. This places the algicidal properties of *P. chrysosporium* in the reported range of algicidal bacteria (70–90%) [29], and at an increased rate. We also used cell counting to quantify the inhibitory effect of *P. chrysosporium* on *M. aeruginosa* at different time points using the growth curves of *M. aeruginosa* cells (Figure 2). We observed that the *P. chrysosporium* had a strong inhibitory effect on *M. aeruginosa*, and the inhibitory effect was time-dependent.

SOD is a protective antioxidant enzyme that is induced during cellular antioxidant defenses and protects cells by scavenging harmful reactive oxygen species (ROS) [30,31]. POD is an integral part of the plant antioxidant system, and plays an important role in plant stress resistance physiology, which has the effect of removes oxygen free radicals and hydrogen peroxide [32]. MDA [33], which is the final product of membrane lipid decomposition, is an important biochemical indicator of oxidative membrane damage, and can reflect the degree of injury suffered by a plant. 

**Figure 3:**
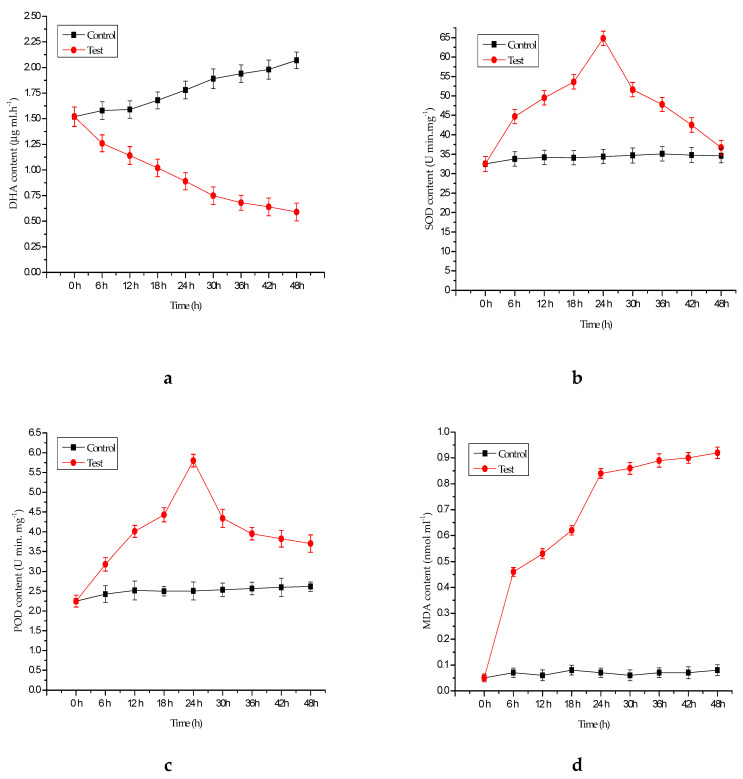
Effects of *P. chrysosporium* on dehydrogenase (DHA) (**a**), superoxide dismutase (SOD) (**b**), peroxidase (POD) (**c**), and malondialdehyde (MDA) (**d**) content in *M. aeruginosa*. Data are expressed as the mean ± SD (*n* = 3).

The reduced DHA levels (Figure 3a) in *M. aeruginosa* and *P. chrysosporium* co-cultures indicates successful degradation of DHA by *P. chrysosporium*, which may, in turn, inhibit chlorophyll-a synthesis and suppress algal growth. The experiments show that the activity of POD and SOD in the experimental groups was significantly higher than that in the control group but sharply declined at 24 h (Figure 3b,c), from 65 U/(min·mg) to 36 U/(min·mg) and from 5.8 U/(min·mg) to 3.7 U/(min·mg), respectively. The MDA level in *M. aeruginosa* co-cultured with *P. chrysosporium* increased to 0.92 nmol·mL^−1^ after 48 h (Figure 3d). These results indicate that *P. chrysosporium* may have caused damage to membranes and chloroplast structural integrity. Such effects suggest that it may be appropriate to use *P. chrysosporium* as an economically sustainable management strategy for controlling harmful algal blooms.

### 3.2. Fourier Transform Infrared Spectroscopy

**Figure 4:**
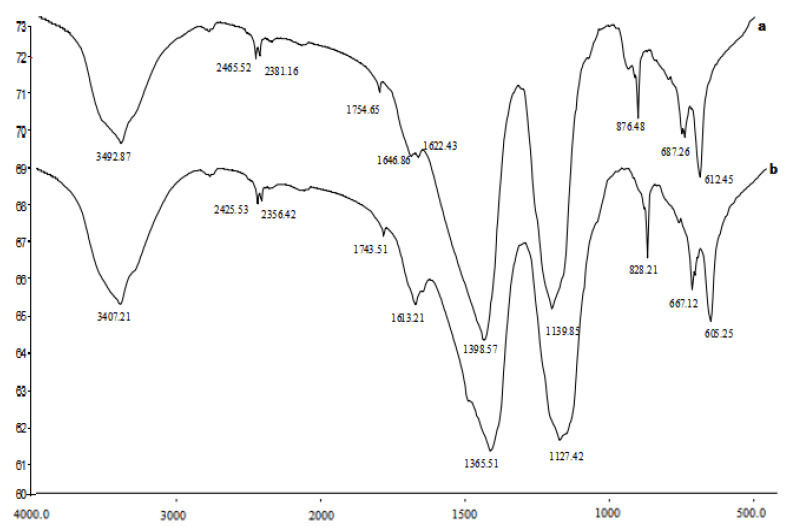
FTIR on *M. aeruginosa*: *M. aeruginosa* alone and (**a**) *M. aeruginosa* treated by *P. chrysosporium* (**b**).

Characterization of the chemical fingerprints of *M. aeruginosa* treated with *P. chrysosporium* and untreated cultures are shown in Figure 4.

The infrared spectrum of the main absorption peak position and the waveform of *M. aeruginosa* cells before and after the white-rot fungi treatment were approximately the same. Irrespective, there is a clear difference among the relative intensities of the peaks. The intensity of the absorption peak of the algal cells treated with white-rot fungi was weaker than that of the untreated algal cells (Figure 4). The algae capture light energy mainly through pigment-protein complexes, and the photosynthetic pigments can be divided into three categories: Chlorophyll-a (Chl), carotenoids (Car), and phycobiliprotein [34]. The absorption at 3100–3500 cm^−1^ was attributed to chlorophyll-a-OH, which stretches adsorbed water molecules. Treatment of *M. aeruginosa* with white-rot fungus altered the absorption peak from 3492.87 to 3407.21cm^−1^, which may be related to the polysaccharides in *M. aeruginosa* cells and damage to the O-H bonds in the algal cell protein components by *P. chrysosporium*. The absorption peak for C-H stretching that is observed at 2300–2900 cm^−1^ changed from the absorption peak 2465.52 to 2425.53 cm^−1^. This may be related to the hydrocarbon bond in the algal cell protein structure being broken by *P. chrysosporium*. The C = O stretching vibration peaks of C_7_ and C_10_·C = O in Chl-a is observed 1600–1700 cm^−1^; however, the intensity of amide C = O stretch of *M. aeruginosa* cells changed from 1646.86 to 1613.21 cm^−1^ after *P. chrysosporium* co-culturing, which indicated that the amide bond of the *M. aeruginosa* cell protein was destroyed. Several sharp small peaks appeared in the range of 600–1000 cm^−1^, indicating disruption of *M. aeruginosa* cell structure and considerable loss of the contents of the algal cells. Finally, the content of chlorophyll-a in *M. aeruginosa* cells decreased. Thus, *P. chrysosporium* showed a high algicidal ability against Chl-a in *M. aeruginosa* and ultimately inhibited the intensity of the algal.

### 3.3. Scanning Electron Microscopy

**Figure 5:**
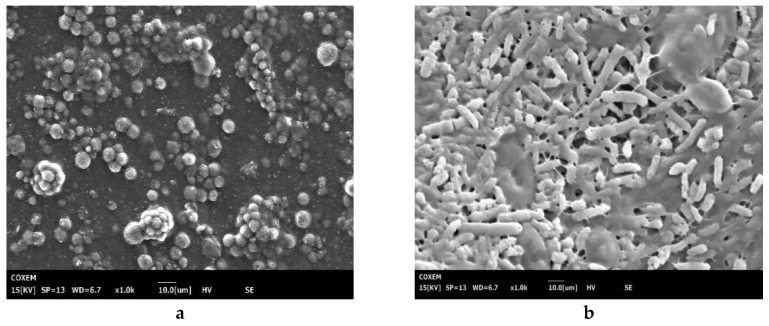
SEM on *M. aeruginosa*: *M. aeruginosa* alone and (**a**) *M. aeruginosa* treated by *P. chrysosporium* (**b**).

SEM can identify changes in the spatial structure of algal cells. Without the *P. chrysosporium* treatment, the *M. aeruginosa* cell structure showed retention of its integrity, strong cell growth, and no intracellular release of soluble substances occurs. Following co-culture with *P. chrysosporium,* the structure of *M. aeruginosa* cells was severely damaged, and soluble cell constituents were released in large quantities (Figure 5). However, from an ecological security perspective, the eco-toxicity of *P. chrysosporium* should be strictly evaluated in future studies before practical applications.

### 3.4. Micronuclei Assay

The algicidal efficiency of fungi on algae was first reported in 2010 [8], but the ecological effects are yet to be reported in detail. The micronucleus test could reflect the damage to and aneuploidy of the chromosome or spindle [35]. Monitoring the micronucleus is a useful technique for determining chromosome damage. Therefore, the micronucleus and frequency of nuclear anomalies can be used as an index to show the genetic toxicity in cells after the physical, chemical and biological processing [36,37].

**Figure 6:**
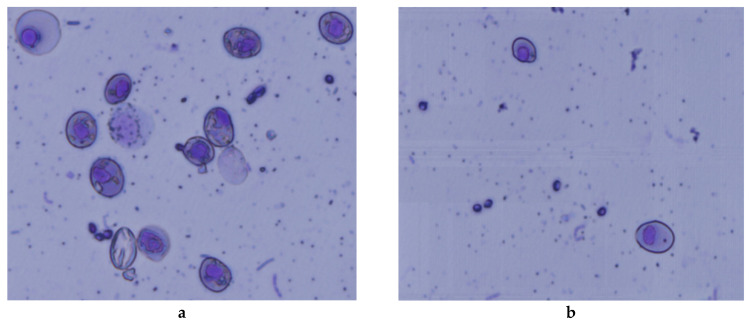
Micronuclei in blood cells of *F. multistriata* tadpoles treated with *M. aeruginosa*: *M. aeruginosa* (**a**) treated by *P. chrysosporium* (**b**).

This study is the first genotoxicity assessment for *M. aeruginosa* as treated with *P. chrysosporium* in the red blood cells of *F. multistriata* tadpoles. According to the results of this study, *M. aeruginosa* could induce micronuclear and nuclear anomalies. Figure 6 shows the visual classifications of the micronucleus. The results showed that the micronucleus and nuclear anomalies rate increased in a certain concentration range. However, the results (Table 1) indicate that the MCN and ONA increased from 1.05 ± 0.09‰ to 4.52 ± 0.07‰ and from 2.45 ± 0.06‰ to 13.21 ± 0.08‰ in the control group treated with algae after 48 h, respectively. Meanwhile, in the *P. chrysosporium* treatment groups, MCN and ONA increase from 1.05 ± 0.09‰ to 1.99 ± 0.05‰ and from 2.45 ± 0.06‰ to 5.83 ± 0.07‰, respectively.

### 3.5. Comet Assay

The effect of environmental pollution on biological genetic material has become a leading concern. The comet assay can quantitatively detect DNA damage in eukaryotic cells, such as single-strand breaks, double-strand breaks and DNA cross-linking [36]. Previous studies have reported no cleavage of DNA cells; moreover, only one circular fluorescent head was noted in a normal blood cell; however, injury to the blood cells led to migration of the fracture fragment towards the anode and formed a tail-like comet as observed the fluorescence microscope [38,39] and could be stained with Trypan blue.

**Figure 7:**
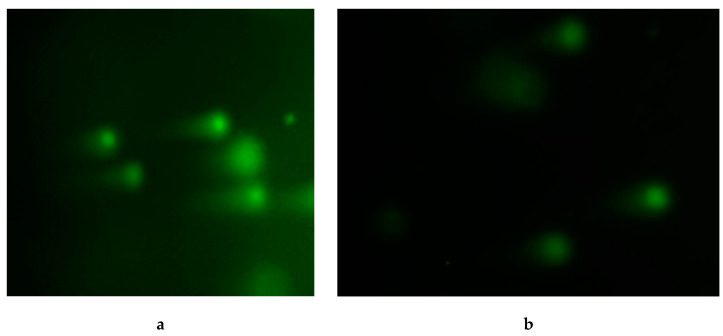
DNA damage in blood cells of *F. multistriata* tadpoles treated with *M. aeruginosa*: *M. aeruginosa* (**a**) treated with *P. chrysosporium* (**b**).

The *F. multistriata tadpoles* were exposed to *M. aeruginosa* or *M. aeruginosa* co-cultured with *P. chrysosporium* for 48 h (Figure 7). Subsequent trypan blue staining showed that the survival of the cells was more than 90% blood cells survived following co-culture treatment, which was consistent with the international standards of the comet assay. For the analysis of the DNA damage in blood cells was analyzed at 0 h, 24 h, and 48 h using the comet test (Table 2). 

The comet assay was performed to analyze the blood cells of *F. multistriata* tadpoles. This test allows the damage induced by high concentrations of algal cells or by algae co-cultured with *P. chrysosporium* to be analyzed. The tail length of the *M. aeruginosa* co-cultured with *P. chrysosporium* was only increased from 1.12 ± 0.21% to 21.68 ± 0.34%, and the comet length was only increased from 6.45 ± 0.09% to 36.45 ± 0.67% within 48 h. The comet rate and degree of DNA damage all decreased compared with the control. This demonstrates that *P. chrysosporium* may inhibit algal blooms and decrease the formation of algal toxins. Although the present study reported the genotoxicity induced by *M. aeruginosa* alone and by *M. aeruginosa* co-cultured with *P. chrysosporium*, much more research needs to be done to improve our understanding of the genetic toxicity of *M. aeruginosa* treated by *P. chrysosporium* in the future, such as the safety for human drinking water.

## 4. Conclusions

The fungus *P. chrysosporium* is a fungal strain that efficiently inhibits and reduces the population of *M. aeruginosa*. Results from the Fourier transform infrared spectroscopy and SEM results suggest that *P. chrysosporium* may destroy the membrane of these algal cells, reduce their photosynthetic intensity, and damage algal structure to inhibit algal blooms. We also found that the number of micronuclei, and frequencies of nuclear anomalies and DNA damage in the red blood cells of *F. multistriata tadpoles* were significantly reduced after treatment with *P. chrysosporium*. Although *P. chrysosporium* can decrease the formation of the algal toxins and has little genotoxicity, many factors influence its ecological safety. Thus, further research is warranted to improve our understanding of the genotoxicity of algae treated with *P. chrysosporium*.

## Figures and Tables

**Table 1:** Micronucleiin blood cells of *Fejervarya multistriata* tadpoles treated with *M. aeruginosa*: *M. aeruginosa* (**a**) treated by *P. chrysosporium* (**b**).

	Time	0 h		24 h		48 h	
Sample							
		MCN ‰	ONA ‰	MCN ‰	ONA ‰	MCN ‰	ONA ‰
Control		1.05 ± 0.09	2.45 ± 0.06	4.31 ± 0.08	10.28 ± 0.10	4.52 ± 0.07	13.21 ± 0.08
Test		1.05 ± 0.09	2.45 ± 0.06	2.13 ± 0.05 **	4.64 ± 0.07 **	1.99 ± 0.05 **	5.83 ± 0.07 **

Compared with control group, ** *p* < 0.01; MCN: micronucleus cell rate; ONA: abnormal cell rate.

**Table 2:** DNA damage induced by *M. aeruginosa* (**a**) treated by *P. chrysosporium* (**b**) to blood cells of *F. multistriata* tadpoles detected by Comet assay.

Time (h)	Tail Length (%)		Comet Length (%)	
0	1.12 ± 0.21 a	1.12 ± 0.21 b	6.45 ± 0.09 a	6.45 ± 0.09 b
24	18.22 ± 0.41 a	10.56 ± 0.45 b **	37.12 ± 0.24 a	21.35 ± 0.32 b **
48	36.41 ± 0.65 a	21.68 ± 0.34 b **	52.36 ± 0.86 a	36.45 ± 0.67 b *

a *M. aeruginosa* (a) and b *M. aeruginosa* (b) treated with *P. chrysosporium*. Compared with control group, * *p* < 0.05; ** *p* < 0.01.
