# Correction: Zeng et al. Algicidal Efficiency and Genotoxic Effects of *Phanerochaete chrysosporium* against *Microcystis aeruginosa*. *Int. J. Environ. Res. Public Health* 2020, *17*, 4029

**DOI:** 10.3390/ijerph22030347

**Published:** 2025-02-27

**Authors:** Guoming Zeng, Maolan Zhang, Pei Gao, Jiale Wang, Da Sun

**Affiliations:** 1 Chongqing Engineering Laboratory of Nano/Micro Biological Medicine Detection Technology, School of Architecture and Engineering, Chongqing University of Science and Technology, Chongqing 401331, China; 2 Institute of Life Sciences & Biomedicine Collaborative Innovation Center, Wenzhou University, Wenzhou 325035, China

In the original publication [1], there was a mistake in Figure 7b as published. The corrected Figure 7b appears below. 

**Figure 7:**
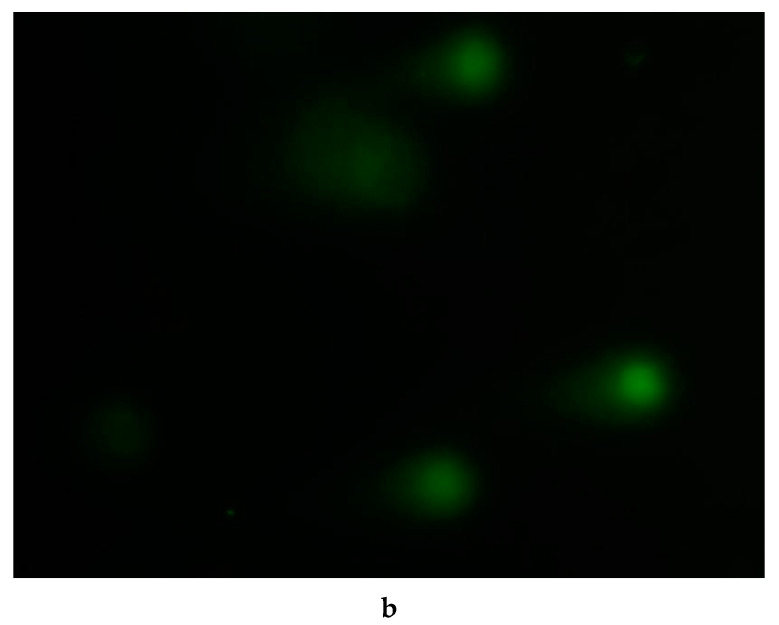
DNA damage in blood cells of *F. multistriata* tadpoles treated with *M. aeruginosa*: *M. aeruginosa* (**a**) treated with *P. chrysosporium* (**b**).

The authors state that the scientific conclusions are unaffected. This correction was approved by the Academic Editor. The original publication has also been updated.
